# Editorial

**DOI:** 10.1007/s41682-020-00062-w

**Published:** 2020-11-03

**Authors:** Heiko Beyer

**Affiliations:** Institut für Sozialwissenschaften, Heinrich-Heine-Universität Düsseldorf, Düsseldorf, Deutschland

‚In Ordnung‘ war die Welt zum Erscheinen der letzten Ausgabe dieser Zeitschrift sicherlich nicht, wie allein der Titel des damaligen Sonderheftes – „Religion und Vorurteil“ – erkennen lässt. Und dennoch scheinen angesichts der globalen Gesundheitskrise, in der momentan Verschwörungstheorien und Bürgerkriegsfantasien gedeihen, die Aussichten zum Ende des Jahres weitaus trüber als noch zu Beginn. Zum aktuellen Zeitpunkt sind über eine Million Menschen weltweit an Covid-19 gestorben, viele Genesene leiden noch an Nachwirkungen und die psychischen Folgen sind kaum zu ermessen. Ebenso dramatisch lesen sich die nun nach und nach erscheinenden sozialwissenschaftlichen Studien zu den gesellschaftlichen Folgen der Pandemie, die von ökonomischen Verwerfungen über politische Destabilisierung bis hin zu digital und analog geführten Kulturkämpfen reichen. Allein anhand der Vielzahl der Ausschreibungen (und eingegangenen Anträge) zu Corona-bezogener Sozialforschung lässt sich die Virulenz der aktuellen Krise ablesen. Diese erstreckt sich auf nahezu alle gesellschaftlichen Bereiche und dürfte noch Jahre, wenn nicht Jahrzehnte die Lebensrealitäten der Menschen, Operationsweisen von Institutionen und Kommunikation in Netzwerken prägen.

Das gilt natürlich auch für das Feld der Religion. Bereits in den letzten sechs Monaten hat sich gezeigt, dass der abgekühlte Krieg zwischen religiösen und wissenschaftlich-säkularen Weltdeutungen wieder aufflammt. So bilden esoterische und neuheidnische Elemente einen wichtigen Teil des ideologischen Fundaments der Impfskeptiker- und Coronaleugner-Szene (Makhashvili und Medeiros 2000; Nguyen und Catalan-Matamoros 2020). Kranz et al. (2000) berichten Ergebnisse einer Untersuchung von 1182 US-Bürger*innen, die erhöhte emotionale Angst sowie eine erhöhte Neigung zu irrationalen Reaktionsweisen (etwa das Vermeiden von 5G-Handynetzen oder Horten von Klopapier) unter religiösen Befragten belegen; selbst unter Kontrolle sozidemografischer Kontrollvariablen erwiesen sich diese Befunde als stabil.

Doch sogar religiöse Individuen und Gruppen, die für wissenschaftliche Erklärungen und moderne Schulmedizin grundsätzlich empfänglich sind, geraten mit zunehmendem Infektionsgeschehen in den Fokus, wenn es um den *praktizierten* Glauben geht: Die gemeinsame religiöse Praxis bildet nicht selten den Kern der religiösen Selbstdefinition und gehört schon deshalb zu den ältesten und wichtigsten Freiheitsrechten moderner Gesellschaften. In der aktuellen Situation jedoch, in der Gottesdienste und religiöse Zusammenkünfte schnell zu Infektionsherden werden können, scheint die Religionsfreiheit plötzlich ein Risiko darzustellen (Quadri 2020). Inzwischen liegen erste Studienergebnisse vor, die bestätigen, was verschiedene Presseberichte der vergangenen Monate erahnen ließen: Höhere Religiosität korreliert mit häufigeren Verstößen gegen Kontakt- und Ausgangssperren (DeFranza et al. 2020). Allerdings erweisen sich religionsbezogene Überzeugungen und Kontakte mitunter auch als wichtige Ressourcen, um die psychischen Folgen der Pandemie abzumildern (Peteet 2000; Pirutinsky et al. 2020). Die Rolle der Religion in der aktuellen Krise dürfte insgesamt vielschichtig, wahrscheinlich sogar paradox sein.

Für ein genaueres Bild bedarf es noch weiterer Untersuchungen. Die zitierten Arbeiten deuten gleichwohl bereits darauf hin, wie eng miteinander verknüpft Religions-, Gesellschafts- und Gesundheitsdiskurse in der gegenwärtigen Lage sind – und vielleicht immer schon waren. Das vorliegende Heft der ZRGP kann zwar noch nicht mit eigenen Beiträgen zu Covid-19 aufwarten, dennoch tragen die hier veröffentlichten Studien dazu bei, den theoretischen und empirischen Rahmen, in dem sich das Verhältnis von Religion und Pandemie derzeit darstellt, klarer abzustecken. Wenngleich die aktuelle Situation ohne Präzedenz ist, erwachsen deren Folgen für den Bereich des Religiösen doch kaum aus dem Nichts.

Führen wir uns mit den Beiträgen dieses Heftes die Grundlagen des Verhältnisses von Religion und Gesellschaft noch einmal vor Augen, so gelangt man zu einem provokanten Resümee, das gleichwohl den im Titel dieser Zeitschrift angedeuteten Zusammenhang nur zu bestätigen scheint: Das Religiöse ist politisch. Und dies selbst da wo es anti- oder a-politisch zu operieren scheint, wie der Eingangsbeitrag von *Detlef Pollack* nahelegt. Pollack betont, dass die klassische differenzierungstheoretische Annahme, der zufolge Säkularisierung vor allem Relativierung des Religiösen bedeutet, konflikttheoretisch zu präzisieren ist. Anknüpfend an Luhmann argumentiert Pollack, dass der gesellschaftliche Differenzierungsprozess der europäischen Moderne vom Überlegenheitsanspruch der Römischen Kirche provoziert worden sei. Die Herausbildung konkurrierender Wertsphären und Teilsysteme, und damit der Bedeutungsverlust der Religion als politische Ordnungsinstanz, entspringe der radikalen Politisierung der christlichen Religion im römischen Episkopat.

Auf das Verhältnis von Religion und Politik, beziehungsweise Religion und Staat, fokussiert auch der Beitrag von *Skadi Siiri Krause*. Dieser zeichnet eine mit historischem Material angereicherte Darstellung der verschiedenen Gestaltformen der Religionsfreiheit, vom pragmatischen Kompromiss der Gewissensfreiheit im 16. und 17. Jahrhundert über das Konzept der Glaubensfreiheit, wie sie im europäischen Kontext des 18. Jahrhunderts zunehmend verhandelt wird, bis hin zur Religionsfreiheit nordamerikanischer Provenienz, die den Schutz religiöser Minderheiten bei gleichzeitiger Trennung von Staat und Kirche in den Mittelpunkt rückt und das Vorbild der meisten Verfassungen des 20. Jahrhunderts darstellt. Angesichts dieser Vielgestaltigkeit des Konzeptes der Religionsfreiheit, die mit der Globalisierung der Menschenrechte seit 1945 noch einmal zugenommen hat und sowohl alte Debatten in neues Licht gerückt als auch neue Fragen aufgeworfen hat – etwa nach der Universalität der ‚westlichen‘ Vorstellung von Religionsfreiheit – kann es nicht verwundern, wie umkämpft das Terrain religionsbezogener Freiheits- und Gleichheitsrechte bis heute ist.

Die folgenden beiden Beiträge des Heftes nähern sich dann dem Thema der Religionsfreiheit über Fallbeispiele religionsbezogener Diskriminierung in der jüngeren Geschichte beziehungsweise der Gegenwart: *Andrei S. Markovits* und *Kenneth Garner* illustrieren zunächst anhand der Erfahrungen jüdischer Studierender im US-amerikanischen Midwest der ersten Hälfte des 20. Jahrhunderts, wie religiöse Minderheiten in einer feindlichen Umwelt ein differenziertes Netzwerk an Organisationen ausbilden, um sich gegen Angriffe der und Ausschlüsse von der Mehrheitsgesellschaft zu wehren. Die Studie beschreibt eindrücklich die unterschiedlichen Wahrnehmungen und Reaktionen der betroffenen Jüdinnen und Juden, die sich mit virulentem Antisemitismus konfrontiert sahen – insbesondere in Michigan, wo mit Henry Ford einer der einflussreichsten US-amerikanischen Antisemiten jener Zeit ansässig gewesen ist. Während „Fraternity and Sorority Jews“ sich an die Mehrheitsgesellschaft und deren Wertvorstellungen anzupassen versuchten, reagierten zionistische und linke Juden, indem sie sich auf jeweils eigene Weise von der amerikanischen Gesellschaft abwanden. Mit *Hillel* existierte darüber hinaus eine explizit jüdische Studierendenverbindung, die gewissermaßen als „catch-all party“ fungiert habe. All diese Gruppen hatten eines gemeinsam: sie zogen vor allem säkulare Jüdinnen und Juden an, keine orthodoxen.

Im Artikel von *Nadine Jukschat* und *Lena Lehmann *fällt der Blick auf Fremdzuschreibungen gegenüber praktizierenden Musliminnen. Auch hier werden unterschiedliche Reaktionsweisen auf Abwertungen durch die Mehrheitsgesellschaft beschrieben. Einige der befragten Musliminnen reagieren mit Strategien der Selbstermächtigung. Diese reichen von ironischen Brechungen verbreiteter Stereotype und der bewussten Demonstration der eigenen Friedfertigkeit bis hin zu aktiven Versuchen, bestehende Vorurteile zu widerlegen. Manche der Interviewten entscheiden sich hingegen für den gesellschaftlichen Rückzug und äußern Resignation angesichts alltäglicher Erfahrungen mit Rassismus.

Der letzte Beitrag des Heftes beschäftigt sich mit einem Diskriminierungsphänomen, bei dem religiöse Akteure häufig eher zur Täter- statt zur Opfergruppe gehören: mit Homophobie. *Kimmo Ketola* und *Eila Helander* untersuchen dabei die Akzeptanz gleichgeschlechtlicher Ehen innerhalb der Evangelisch-Lutherischen Kirche Finnlands (ELCF). Als Resultat halten sie fest, dass sowohl die Mehrheit der Bevölkerung als auch der Mitglieder der ELCF die gleichgeschlechtliche Ehe befürworten. Dies treffe jedoch nicht auf die Entscheidungsträger innerhalb der ELCF zu. Dieser Meinungsunterschied zwischen allgemeiner Bevölkerung, Mitgliedern und Verantwortlichen sei vor allem darauf zurückzuführen, dass letztere im Durchschnitt älter und religiöser seien, und ältere und religiösere Personen tendenziell zu den Gegner*innen der gleichgeschlechtlichen Ehe gehören.

Wenn auch die hier versammelten Artikel damit nicht nur thematisch und regional, sondern auch konzeptuell sehr heterogen sind, haben sie doch, wie erwähnt, eines gemeinsam: sie belegen mit unterschiedlichstem Material, wie eng verwoben religiöse und politische Kämpfe historisch waren und heute noch sind. Zu vermuten steht, dass diese Kämpfe in der aktuellen Situation an Fahrt aufnehmen, verspricht religiöse Zugehörigkeit doch einen sicheren Hafen im Sturm der Krise. Die autoritäre Rede von der ‚Verteidigung des christlichen Abendlands‘, die seit dem Ausbruch der Pandemie nicht nur lauter, sondern auch ‚bunter‘ geworden ist, mag häufig nur instrumentelle Semantik sein, die nicht mit individuellen religiösen Überzeugungen korrespondiert. Aber ihre Forderung nach der Ausgrenzung oder sogar Vernichtung der religiös Anderen, vor allem von Juden und Muslimen, ist durchaus ernst gemeint. Gleichzeitig wird sehr wahrscheinlich der Staat aufs Neue die Religionsfreiheit (wie auch andere Freiheiten) einschränken müssen, wenn die Gesundheitslage dies zu erfordern scheint. Für eine Gesellschaft, die sich ganz wesentlich über ihre Grundrechte definiert, muss dieses Dilemma auf lange Sicht Folgen für die Legitimität des politischen Systems haben. Die sozialwissenschaftliche Religionsforschung kann dieses Dilemma zwar nicht lösen, aber zumindest zu seiner theoretischen Entfaltung und empirischen Bestimmung beitragen.
